# Metabolites produced by inoculated *Vigna radiata* during bacterial assisted phytoremediation of Pb, Ni and Cr polluted soil

**DOI:** 10.1371/journal.pone.0277101

**Published:** 2022-11-10

**Authors:** Uzma Zulfiqar, Azra Yasmin, Anila Fariq

**Affiliations:** 1 Department of Environmental Sciences, Fatima Jinnah Women University, Rawalpindi, Pakistan; 2 Department of Biotechnology, Fatima Jinnah Women University, Rawalpindi, Pakistan; Benemérita Universidad Autónoma de Puebla: Benemerita Universidad Autonoma de Puebla, MEXICO

## Abstract

Phytoremediation assisted with plant growth promoting bacteria (PGPB) is a green technology to remediate metal contaminated soils. Plants usually produce secondary metabolites to tolerate metal toxicity. Present study was designed to explore the phytoremediation potential of *Vigna radiata* var. NM-II in the presence of metal resistant PGPB and comparison of metabolites produced under heavy metal stresses (Pb, Ni, Cr). Three PGPB selected for present study include *Bacillus pumilus* MB246, *Serratia nematodiphila* MB307 *and Delftia Lacustris* MB322. Pot experiments were conducted with inoculated *V*. *radiata* NM-II seeds grown in soil artificially contaminated with lead (Pb), Nickle (Ni) and chromium (Cr) at a concentration of 300, 200 and 100 mg/kg respectively. After harvesting various growth parameters were studied (root length, shoot length, fresh weight and dry weight). Bacterial colonization on root surfaces of harvested plants was observed through Scanning electron microscopy (SEM) and Elemental composition was recorded through Energy dispersive X-ray spectroscopy (EDX) attached with SEM. Metabolic response of harvested plants was studied through Gas chromatography Mass spectrophotometry (GC-MS) analysis. Metal accumulation in roots, shoots and soil was analysed by acid digestion method from which Bioaccumulation factor (BF) and Translocation factor (TF) of metal from soil to plant was calculated. Results revealed stimulatory effect of PGPB on growth and phytoextraction ability of *V*. *radiata*. Soil metal removal efficiency was in the order Pb>Ni>Cr, whereas metal distribution in each part of plant was root>stem>leaf. The BF and TF values suggested *V*. *radiata* as Pb and Ni excluder while moderate accumulator for Cr. Elemental analysis through Energy Dispersive X- ray spectroscopy (EDX) found potassium (K^+^)and calcium (Ca^+^)as highly abundant nutrients with least accumulation of sulphur (S). Metabolites study through GC-MS revealed variety of compounds (carbohydrates, amino acids, fatty acids, steroids etc) detected differentially under each metal treatment and their concentration was influenced by different bacterial inoculations. Overall 9-Octadecenamide was found as commonly present lipid compound in most of the treatments which is required for detoxification in plants. The study concluded beneficial role of PGPB for successful phytoremediation of heavy metals and differential response of metabolites towards each metal stress that is related to metal tolerance ability of *V*. *radiata*.

## Introduction

Lead (Pb), nickle (Ni) and chromium (Cr) are considered as the major heavy metal (HM) pollutants, which negatively affect all forms of life. Phytoremediation assisted with plant growth promoting bacteria (PGPB) is an attractive strategy for remediation of heavy metal contaminated soils. Metal-resistant PGPB *Enterobacter cloacae*, *Bacillus cerus*, *Pseudomonas putida*, *Microbacterium oxydans*, *Pseumdomonas sis*, and *Burkholderia cepacia* were found effective in promoting the phytoremediation of metal polluted soil in several studies [1–5].

Some limitations in the use of hyperaccumulator plants is slow growth rates and less biomass production that can be overcome by the use of fast growing and high biomass producing crop. Studies on crop plants are also important because of the economic perspective as the harvestable biomass has nutritional value to meet the energy requirements of large population and can be used as source of bioenergy. Legumes have been proposed for phytoremediation purpose due to legume rhizobia interaction [6]. They are ideal candidates to adapt in moderately metal polluted soils. Legumes mostly behave as metal excluders due to higher accumulation of metals in the roots having low translocation to the aboveground parts of plant [7]. Among legumes, *V*. *radiata* is reported for its high metal tolerance and metal accumulation ability [8–12]. It is a short duration crop with vigorous growth and can grow in various agro ecological zones with diverse cropping systems and practices. Keeping in view the importance of leguminous plants and their growth potential in metal stress environment we selected *Vigna radiata*, as less work is being done on its use as phytoaccumulator as compared to other crops.

Plant growth-promoting bacteria (PGPB) associated with leguminous plants may contribute to the absorption, growth, metal tolerance and acceleration of the phytoremediation process. *Pseudomonas putida* has been reported successfully for enhancing the growth of *V*. *radiata* under metal stress conditions [13]. Several other studies reported enhanced growth of inoculated *V*. *radiata* in the presence of metals [14, 15].

Plants used for successful phytoremediation can tolerate and accumulate large amount of metals in their above ground parts by producing secondary metabolites. Metal stress resulted in alterations in the metabolic pool of plants to channelize the production of new biochemically related metabolites which help in metal tolerance [16]. The main purpose of metabolic profiling is to extract, separate and analyse a spectrum of metabolites [17]. Various kinds of secondary metabolites are formed by plants under metal stress. Metal stress tolerance in plants can be improved by manipulating the biosynthesis and accumulation of secondary metabolites [18]. GC-MS analysis of Subabul plant biomass intended for the phytoremediation of soil contaminated with dye revealed the presence of tetradecyne, palmatic acid, pelargonic acid, pyridine, myristic acid and oxalane as active compounds [19]. Some amino acid and organic acid ligands play an important role in transportation and homeostasis of HMs in plants e.g. metallothioneins (MTs) and phytochelatins (PCs). They bind HMs inside the cell which helps in sequestration and detoxification process [20].

The metabolic analysis of radish roots under Pb and Cd stress revealed alterations in sugars, organic acids and amino acids [21]. These metabolites play a role as osmo-protectants, antioxidants and by-products of stress as signal transduction molecules in stress conditions [22]. Organic acids increase bioavailability of heavy metals by reducing pH around the rhizosphere which resulted in activation of insoluble minerals in soils [17]. According to Debela et al. [23] Oxalic acid can play a role in release of Pb from soils contaminated with pyromorphite. Betalains content in *Beta vulgaris L*. plant was increased in the presence of Cu [24]. In plants citrate is the most abundant carboxylic acid which acts as a ligating agent for hyperaccumulation of Ni [25]. Metabolic analysis in Bermuda grass revealed seven amino acids (glycine, glutamic acid, threonine, proline, norvaline, gluconic acid, and serine), three sugars (galactose, xylulose, talose) and four organic acids (oxoglutaric acid, citric acid, malic acid, andglyceric acid) as highly abundant compounds in variety WB242 [26]. In Aquatic duck weed, lead mediated bioaccumulated biomass showed the presence of Hexadecenoic acid, Nonadecanic acid, Benzoic acid, Phthalic acid, Trans13-octadeconic acid, Sulfurous acid, Methoyacetic acid and 2-Acetylbenzoic acid.

The nutrient elements phosphorus (P), sulphur (S) and potassium (K) are known to play an important role in growth and metabolism of plants. They contribute towards survival of plants under various biotic and abiotic stresses [27, 28]. HMs severely inhibits the growth of plants and even resulted in plant death by disturbing the nutrients uptake [29]. Several studies have reported reduction in uptake of most mineral elements e.g Ca, Mg, K and Fe under HM stress [30–32]. Rhizospheric plant growth promoting bacteria are useful in solubilizing, mobilizing, and transforming nutrients. They improve the availability of minerals through various direct and indirect mechanisms, including atmospheric nitrogen fixation, phosphate and potassium solubilization and by releasing certain volatile organic compounds [33]. The selected PGPB in present study have the capacity to promote growth of *V*. *radiata* under Pb, Ni and Cr stress and enhance the phytoextraction of these metals from soil. They can also promote the production of secondary metabolites to mitigate the effect of metal stress. The objectives of current study were to assess the impact of PGPB on phytoremediation ability of *V*. *radiata* to remediate Pb, Ni and Cr polluted soil and to identify the predominant metabolites produced in response to metal stress using GC-MS analysis. Current study has novelty and practical applications which are of relevance to food, crops, biodiversity and climate change and will have true value to the public. The study is supportive for agriculturist and environmentalist to clean the agricultural land from metal pollution without any extra cost and make it productive for growth of crops.

## Materials and methods

### Pot experiment

Pot experiments were conducted in a greenhouse located at Fatimma Jinnah Women University, Rawalpindi, Pakistan under natural conditions in the month of April. The geographical description of study area is Latitude: 33.6007, Longitude: 73.0679, 33° 36’ 33” North, 73°4’ 44” East. Altitude is 497 m with humid subtropical climate. The average temperature was 26°C and the photoperiod was 12 hrs.

Garden soil used for this study (0–20 cm depth) was first air dried and sieved (2 mm). It was then analysed for various physiochemical characteristics. Soil pH and electrical conductivity (EC) were determined in 1:1 soil to water suspension [34]. For the measurement of organic matter, loss on ignition method was used [35]. Moisture content was determined by drying method. For phosphate and sulphate analysis methods of Mussa et al. [36] were used.

Soil was sterilized and artificially contaminated with Pb, Ni & Cr. Metals were added in soluble form to ensure plant availability. Aqueous solutions of PbNO_3_, NiCl_2_ and K_2_CrO_4_ were prepared in autoclaved distilled water to provide a contamination level of 300, 200 and 100 mg of Pb, Ni and Cr kg^–1^ of soil, respectively. These solutions were poured on soil and mixed thoroughly for even distribution of each metal. Soil was then air dried in shade and allowed to settle for 2–3 days.

Small sized clay pots (5x6 inches) were used for plant growth. Each pot was filled with half kg of soil. Three strains were selected for seeds’ inoculation based on their performance in previous hydroponic experiments [37]. These include *Bacillus pumilus* MB246, *Serratia nematodiphila* MB307 *and Delftia Lacustris* MB322. Seeds of *V*. *radiata (var*. *NM-II)* were obtained from National Agricultural Research Centre, Islamabad, Pakistan. Healthy and surface sterilized seeds (with 0.1% HgCl_2_) were inoculated with their respective single bacterial suspension. For bacterial suspension twenty four hour freshly prepared bacterial cultures grown on Nutrient agar medium supplemented with 200 μg/ml of respective metal (i.e. Pb, Ni and Cr) was used. Inoculum was prepared by mixing bacterial culture in 10 mL autoclaved distilled H_2_O. The optical density of inoculums was adjusted to 10^6^ cells per mL at 600 nm in spectrophotometer in order to give uniform inoculum of each strain.

Control seeds were soaked in autoclaved distilled H_2_O. Initially eight seeds were sown in pot soil and irrigated with water. After seedlings germination, each pot was thinned to five plants per pot. Plants were supplied with water and Nutrient solution [38] as required and allowed to grow under natural conditions. After three weeks of growth plantlets were removed carefully from the pots and washed. Growth was measured in terms of root length and shoot length. Plantlets were separated into roots, stems and leaves and their fresh weights were recorded individually. Some plant material was frozen for metabolic studies while remaining was dried at 70°C for 24 hrs.

### Heavy metal analysis

Heavy metal analysis was done by acid digestion method. Dried plant material (0.5g) was ground and digested with a mixture of HCl/HNO_3_ (3:1, v/v). Concentrations of metals in the digested samples were determined using atomic adsorption spectroscopy (AAS). Bioaccumulation factor (BAF) was calculated by dividing the total concentration of metal in plant tissue by the total concentration of metal in targeted soil.


$$\text{Bioaccumulation}\;\text{factor}\;\left(\text{BF}\right)=\frac{\text{Average}\;\text{conc.}\;\text{of}\;\text{metal}\;\text{in}\;\text{the}\;\text{whole}\;\text{plant}\;\left(\text{mg}/\text{kg}\right)}{\text{Metal}\;\text{added}\;\text{in}\;\text{soil}\;\left(\text{mg}/\text{kg}\right)}$$

Translocation Factor (TF) i.e. ratio of HMs concentration in plant shoots to its roots was calculated according to Yadav et al. [39].


$$\text{Translocation}\;\text{factor}\;\left(\text{TF}\right)=\frac{\text{C}\;\text{aerial}}{\text{C}\;\text{root}}*100$$

### Efficiency of metal removal from soil

Percentage removal of metal from soil was calculated as follows;

$$\%\text{removal}=\frac{\left[\text{Co}-\text{Ce}\right]}{\text{Co}}\times 100$$

Co = initial metal concentration in soil
Ce = final metal concentration in soil

### Bacterial colonization on root by Scanning Electron Microscopy (SEM)

To study the bacterial colonization on the surfaces of plant roots, the sterilized *V*. *radiata* seeds were first germinated and grown in sterilized petri plates. After the growth of seven days in petri plates these seedlings were then allowed to grow in 50 mL tubes each supplied with 25 mL of Hoagland’s nutrient solution and 100 *μ*g/mL of respective metal solution (Pb, Ni and Cr). The suspended seedlings in each tube were inoculated with one mL of their respective bacterial suspension. Seven days old seedlings were harvested. Some samples were frozen for GC-MS analysis. For scanning electron microscopy, harvested plant roots were dried and then cut into small pieces of 5–10 mm size. These pieces were mounted onto metal stub with double sided carbon tape and sputter coated with gold palladium. Each sample was observed under SEM (Tescon model) at varying magnifications (500, 1000 and 5000) with 20KV light. Energy dispersive X-ray spectroscopy (EDX) attached with SEM was used for the identification of elemental composition of samples. The atomic percentage of elements in each plant sample was obtained by X-ray emission based spectral peaks.

### Metabolite profiling by Gas chromatography Mass spectrometry (GC-MS)

GC-MS is used for quantitative and qualitative detection of compounds present at molecular levels with very high accuracy. In current study the metabolic analysis was carried out by Schimadzu Gas Chromotograph and Mass-spectrometer following the method of Sun et al. [40]. For metabolite extraction, 100 mg of harvested frozen and ground plant material was extracted in 5 mL of extraction mixture (methanol/chloform/water in 2.5:1:1v/v/v) for 2 hrs by continuous shaking at room temperature. The extraction solution was centrifuged at 15000 rpm for 10 minutes. The polar supernatant (methanol/water) phase and non-polar (chloroform/methanol) phase were collected in separate tubes. The polar phase was reduced to dryness and dried polar residue was methoximated with 50 *μ*L of 20 mg/mL of methoxyamine hydrochloride in pyridine for 90 min at 37°C followed by trimethylsilylation with 80 *μ*L *N*-methyl-*N*-trimethyl silyl triflouroacetamide (MSTFA) for 60 min at 37°C. For metabolite analysis, 1 *μ*l of derivatized sample was injected in GC-MS.

Non-polar layer was used for lipid analysis. For this samples were resuspended in 0.4 mL chloroform followed by addition of 0.5 mL of 0.25M HCl in methanol and incubated at 50°C for 4 hrs. After evaporation of solvents and HCl, the samples were resuspended in 35 *μ*L of pyridine and again incubated at 50°C until all the residue dissolves. Samples were then treated with 30 *μ*L of MSTFA and incubated at 50°C for 1 hr. 1 *μ*L of sample was injected in GC-MS for lipid analysis. All compounds were identified by using mass spectrum data of GC-MS coupled with National Institute Standard and Technology (NIST) database attached with GC-MS and from NIST chemistry web book.

### Statistical analysis

All measurements were taken as mean of three replicates ± standard error of means. Metabolites Data was analysed by Principal component analysis using PAST software.

## Results

The results of various physiochemical characteristics of soil are displayed in S1 Table in S1 File. It was a light brownish soil was sandy loam texture. It has a 9 pH and electrical conductivity 144 (μs/cm), Moisture content of the soil was 1.62%, while it contained 2.11% organic matter.

### Growth response of *Vigna radiata* in Pb, Ni and Cr contaminated soil

The growth response of *V*. *radiata* with respect to length (root and shoot) and weight (fresh and dry) were studied and displayed in Table 1. It was observed that all the growth indicators in inoculated *V*. *radiata* were higher than uninoculated control. The growth response also varied with different bacterial inoculations. Highest increase in root length was observed with MB246 (15.97%) inoculation followed by MB307 (12.2%). However, the shoot length exhibited maximum increase with MB307 inoculation (11%) followed by MB322 (10.7%). In case of fresh weights and dry weights, maximum increase was observed with MB322 inoculation i.e. 29.1% and 23.3% respectively.

**Table 1:** Physiological growth response of *Vigna radiata* under heavy metal stress.

Bacterial strains	Root Length	Shoot Length	Fresh weight	Dry weight
	(cm)	(cm)	(g)	(g)
**Control**	2.63±.22	21.39±1.5	1.96±.43	0.30±.11
**MB246**	3.05±.09	23.64±1.4	2.23±.43	0.34±.07
**MB322**	2.69±.1	23.68±1.6	2.53±.55	0.37±.02
**MB307**	2.95±.14	23.74±1.0	2.27±.42	0.31±.04

Results are mean of triplicate experiments ±Standard error of mean

### Metal remediation performance of *Vigna radiata* towards Pb, Ni and Cr

**Fig 1:**
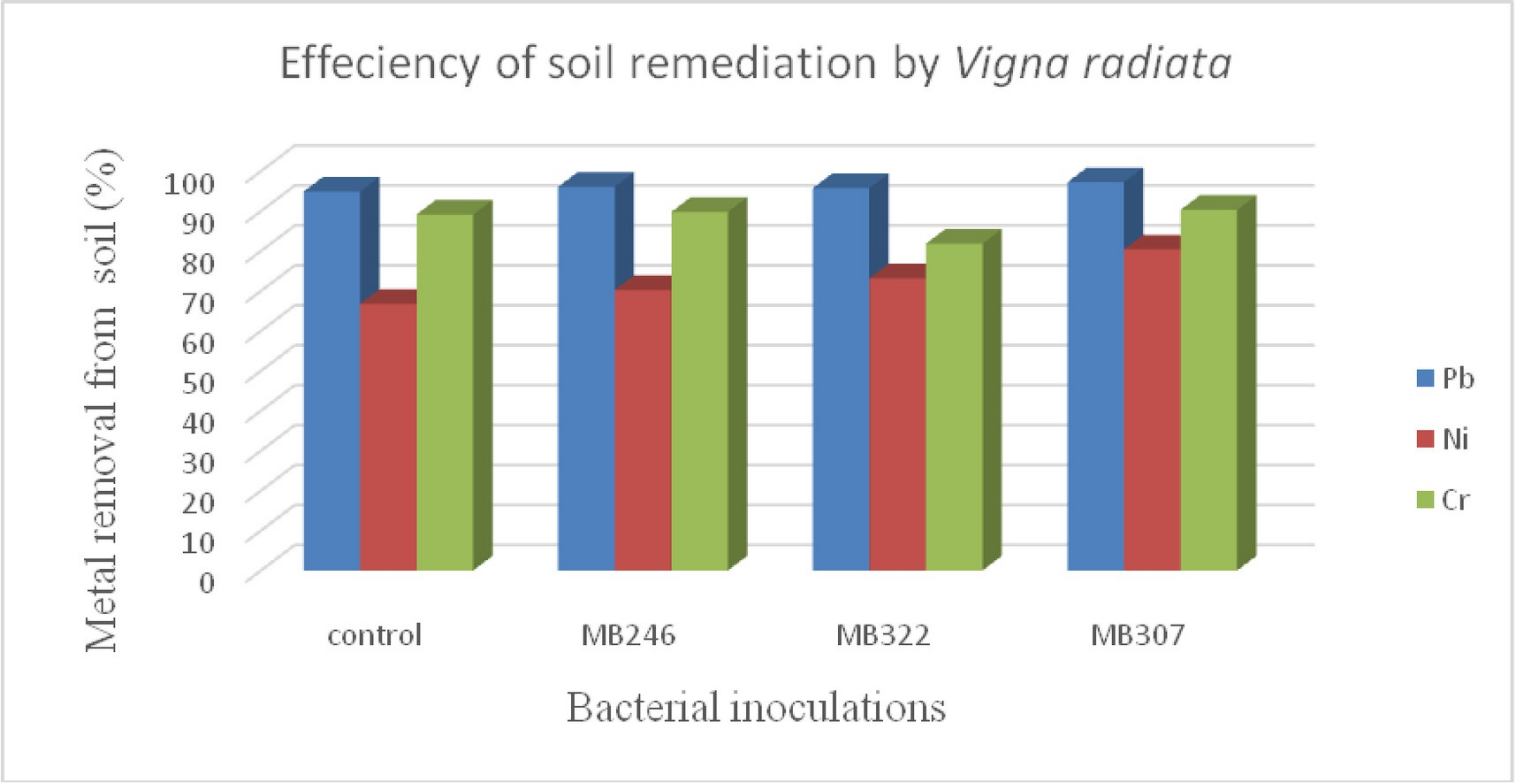
Comparison of metal removal efficiency of *Vigna radiata* under each treatment.

The performance for phytoremediation of Pb, Ni and Cr by *V*. *radiata* was evaluated through its Bioaccumulation Factor (BF) and Translocation factor (TF) values as shown in Table 2. It was observed that metal uptake by plant proportionate to its concentration in the soil. It increased with the increase in metal concentration. The BF and TF values varied with each metal. Maximum BF values were observed for Cr followed by Ni and Pb. However, Cr exhibited minimum TF values. Bacterial inoculations also variably affected metal uptake. Maximum BF for Ni and Cr was with MB246 inoculation i.e. 0.6 and 0.71 respectively. Whereas, for Pb maximum BF was observed with MB307 inoculation i.e. 0.52. Results of TF for all metals revealed maximum values with MB322 inoculation i.e. 0.99 for Pb followed by 0.93 for Ni and 0.36 for Cr. The metal remediated from soil was determined by analysis of metal left in soil after harvesting (Table 3). The efficiency of metal removal from soil (Fig 1) showed Pb as highly remediated metal followed by Cr and Ni.

**Table 2:** Bioaccumulation and translocation factors of *Vigna radiata* grown in soil amended with different concentrations of Pb, Ni and Cr.

Bacterial Strains	Bioaccumulation factor (BF)			Translocation factor (TF)		
	Pb	Ni	Cr	Pb	Ni	Cr
**Control**	0.50±.02	0.48±0.16	0.62±0.01	0.53±0.03	1.06±0.27	0.17±0.02
**MB246**	0.42±.08	0.60±0.20	0.71±0.12	0.98±0.13	0.76±0.27	0.12±0.03
**MB322**	0.41±.06	0.50±0.13	0.63±0.02	0.99±0.12	0.93±0.22	0.36±0.02
**MB307**	0.52±.01	0.53±0.12	0.66±0.03	0.73±0.05	0.90±0.18	0.30±0.02

Results are mean of three values ±Standard error of mean

**Table 3:** Remaining metal in soil (mg/g) after harvesting of inoculated *Vigna radiata*.

Bacterial inoculations	Pb	Ni	Cr
**Control**	0.77±0.13	3.32±0.63	0.54±0.42
**MB246**	0.59±0.15	2.98±1.02	0.51±0.39
**MB322**	0.64±0.05	2.69±0.31	0.91±0.50
**MB307**	0.42±0.21	1.96±0.84	0.49±0.40

Results are mean of three values ±Standard error of mean

### Distribution of Pb, Ni and Cr in different parts of *Vigna radiata*

**Fig 2:**
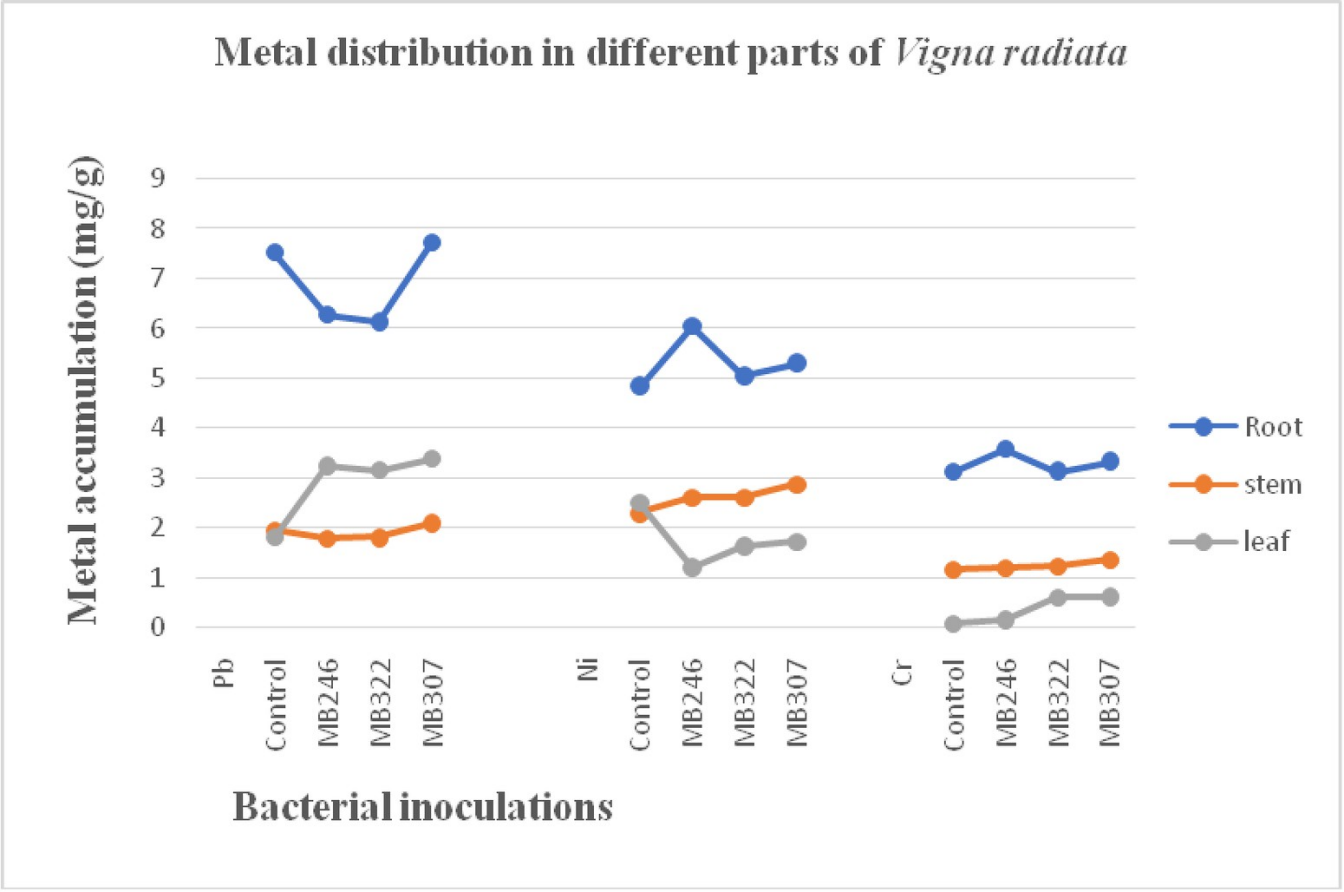
Dispersal of metals in root, stem and leaf of *Vigna radiata* grown in soil contaminated with different concentrations of Pb, Ni and Cr.

Concentration of metal that accumulated in each part of *Vigna radiata* was studied through atomic absorption spectroscopy. Metal uptake by root, stem and leaf varied towards each metal and with each bacterial strain (Fig 2). Overall maximum accumulation of metals was observed in roots as compared to shoots. Metal content accumulated in each part of *V*. *radiata* decreased in the order; root>stem>leaf. However, the trend slightly changed with Pb having more accumulation in leaf as compared to stem. The highest metal concentration in root, stem and leaves of *V*. *radiata* were found in MB307 inoculated plantlets for Pb i.e. 3.38mg/g, 1.73mg/g for Ni in stem and 0.63mg/g for Cr in leaf.

### SEM-EDX studies

**Fig 3:**
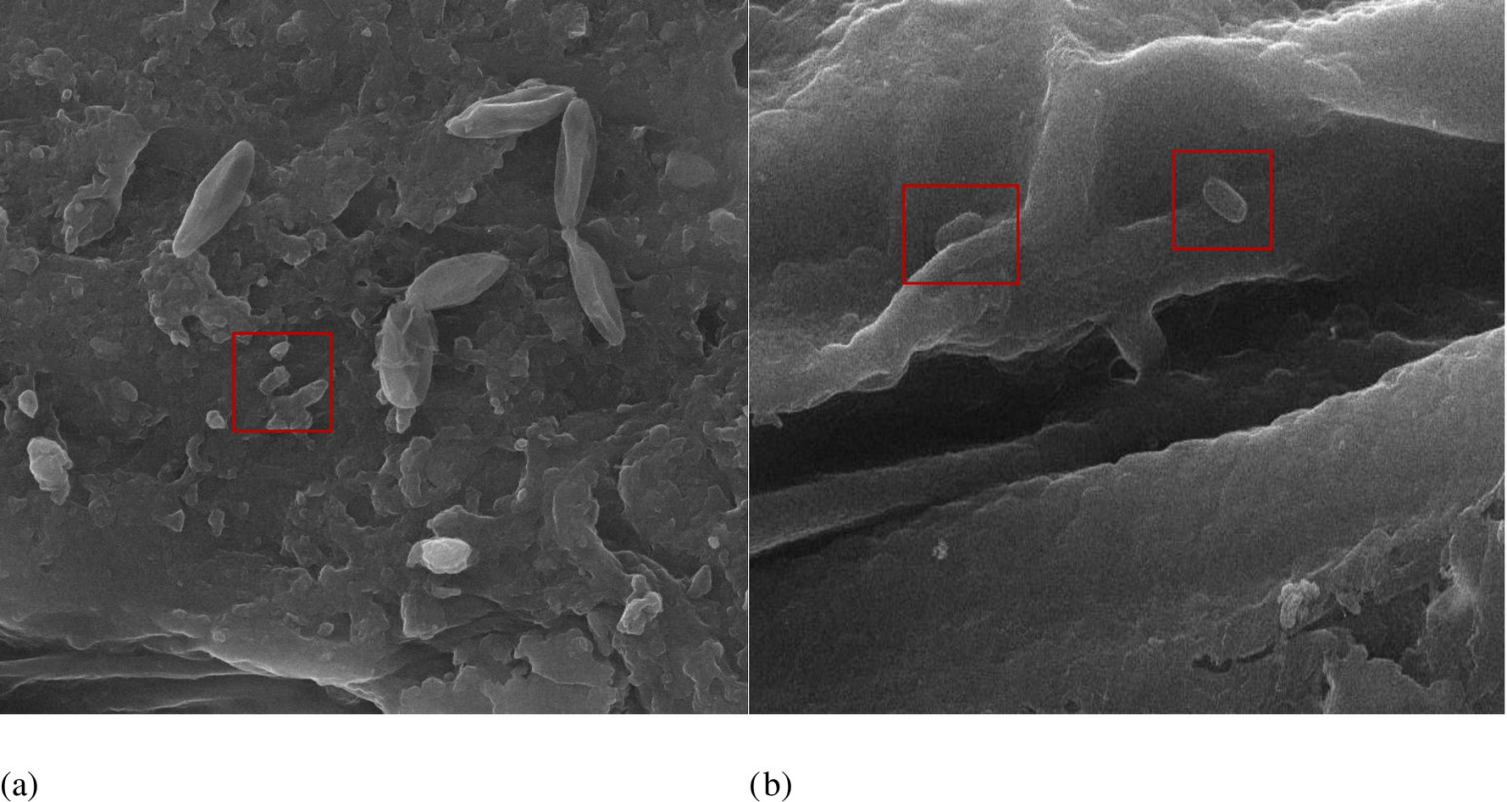
Scanning electron micrographs of inoculated *Vigna radiata* seedlings showing bacterial colonization at surface (a) MB246 (b) (307).

Scanning electron microscopy of inoculated *V*. *radiata* seedlings showed presence of bacterial strains on root surfaces as seen in Fig 3. Energy Dispersive X-ray (EDX) attached with SEM was used for elemental analysis. Elemental profile (weight %) in different parts of plant is displayed in Table 4. Potassium (K) was recorded as highly abundant element in most of the samples whereas Sulphur (S) was found in minimum percentages. Highest concentration of Ca, K and Mg was found in MB307 inoculated stem. Whereas, Al, Fe and Si were present most abundantly in MB246 inoculated roots.

**Table 4:** Elemental composition (percentages) in different parts of *Vigna radiata*.

Plant samples	Elements (weight %)							
	Mg	S	Cl	K	Ca	Fe	Al	Si
**Control, root**	0.37	0.28	0.70	1.81	1.61	0.45	0.64	1.39
**Control, stem**	0.25	0.55	0.51	2.12	1.91	0	0	0.64
**Control, leaf**	0.3	0	0.52	1.53	1.57	0	1.2	0.94
**MB246, root**	1.52	0	0	2.81	4.35	5.58	5.86	18.9
**MB246, stem**	1.77	0.82	2.44	9.83	5.23	2.26	1.48	4.41
**MB246, leaf**	0.6	0	0.35	1.57	3.2	1.88	1.6	4.24
**MB322, root**	1.8	0	2.93	7.39	4.44	2.08	2.44	4.81
**MB322, stem**	1.51	0.38	3.12	11.6	5.29	2.35	0	2.62
**MB322, leaf**	0.67	0.29	0.62	1.77	2.26	1.06	1.3	3.38
**MB307, root**	1.71	0	0.78	2.78	4.54	5.35	5.86	18.43
**MB307, stem**	2.08	0	2.87	11.34	5.37	0.11	1.13	3.08
**MB307, leaf**	0.78	0	0.27	1.2	1.41	1.86	1.12	2.51

### Metabolic response of *V*. *radiata* to metal stress

Metabolites present in plant extracts of uninoculated and inoculated *V*. *radiata* seedlings exposed to 100 *μ*g/mL of Pb, Ni and Cr was studied through GC-MS. Both polar and non-polar phases of plant extracts were analysed to study the variety of lipids and other secondary metabolites present in these layers. Name of compounds along with their molecular formula, concentration (peak area %) and retention time (RT) are displayed in Tables 5 and 6 respectively. Identified compounds were analysed through principal component analysis (PCA).

**Table 5:** Compounds identified in lower lipophilic extract (non polar) layer of *Vigna radiata*.

Sample	RT	Compound3	Molecular formula	Peak Area %
**Control+ 0**	33.64	8,11,14-Eicosatrienoic acid, methyl ester	C21H36O2	7.66
	30.25	Pentadecanoic acid, 14-methyl-, methyl ester	C17H34O2	4.13
**MB246+ Pb 100**	33.36	Picolinamide	C6H6N2O2	3.32
	30.23	Hexadecanoic acid, methyl ester	C17H34O2	3.11
**MB322+ Pb 100**	35.35	Nonadecanoic acid	C_19_H_38_O_2_	1.74
	33.53	6-octadecenoic acid, methyl ester	C19H36O2	12.87
	30.23	Pentadecanoic acid	C17H34O2	8.68
**MB307+ Pb 100**	46.04	cis-13-Eicosenoic acid, picolinyl ester	C_26_H_43_NO_2_	0.21
	39.63	l-Cysteine, N,S-bis(2,6-difluorobenzoyl)-, methyl ester	C_18_H_13_F_4_NO_4_S	1.16
	34.58	11,14,17-Eicosatrienoic acid, methyl ester	C21H36O2	6.45
	30.25	Sarcosine, N-isobutyryl-, tetradecyl ester	C_21_H_41_NO_3_	0.12
**MB246+ Ni 100**	44.67	8-Methyl-6-nonenamide	C10H19NO	2.04
		Sarcosine, N-(3-bromobenzoyl)-, butyl ester	C_14_H_18_BrNO_3_	
	35.37	Hexadecanamide	C16H33NO	8.19
		4-Dimethylamino-3,5-dinitrobenzoic acid	C_9_H_9_N_3_O_6_	
	33.63	Picrolonic acid	C_10_H_8_N_4_O_5_	17.84
	20.63	L-Glutamic acid	C_5_H_9_NO_4_	8.73
	36.0	Sarcosine, N-(2-trifluoromethylbenzoyl)-, butyl ester	C_15_H_18_F_3_NO_3_	14.49
	34.45	6-Octadecenoic acid, methyl ester	C19H36O2	44.1
	30.94	Hexadecanoic acid, methyl ester (palmitic acid, methyl ester)	C17H34O2	22.82
	21.83	Glycolic acid, 2TMS derivative	C_8_H_20_O_3_Si_2_	3.29
**MB307+ Ni 100**	39.53	Cyclopropanecarboxylic acid,-2-(2-propynyl) methyl ester	C8H10O2	4.64
		Docosanoic acid	C_22_H_44_O_2_	
	34.47	6-Octadecenoic acid	C19H36O2	17.3
	36.33	Sarcosine, N-(2-trifluoromethylbenzoyl)-, butyl ester	C_15_H_18_F_3_NO_3_	3.44
	30.92	Hexadecanoic acid, methyl ester (palmitic acid, methyl ester)	C17H34O2	6.03
	7.78	Glycine, N-(phenylacetyl)-, methyl ester	C_11_H_13_NO_3_	54.63
**MB246+ Cr 100**	34.42	11-Octadecenoic acid, methyl ester	C19H36O2	8.84
	30.93	Hexadecanoic acid, methyl ester	C17H34O2	5.15
	5.47	L-Glutamic acid	C_5_H_9_NO_4_	57.8
**MB322+ Cr 100**	30.54	Nickel, (eta-2-2-diallyl ether)	C11H15NNiO	0.2
	30.96	Pentadecanoic acid	C17H34O2	4.52
	34.44	9-Octadecenoic acid, methyl ester	C19H36O2	9.89
	41.42	cis-5,8,11-Eicosatrienoic acid, methyl ester	C_21_H_36_O_2_	0.84
	6.48	Glycine, N-(phenylacetyl)-, methyl ester	C_11_H_13_NO_3_	37.56
**MB307+ Cr 100**	41.46	9-Octadecenoic acid, methyl ester	C19H36O2	4.54
	36.35	Tetradecanamide	C14H29NO	8.6
	30.93	Hexadecanoic acid, methyl ester	C17H34O2	16.09

**Table 6:** Compounds identified in upper methanolic extract (polar layer) of *Vigna radiata*.

Sample	RT	Compound	Mol. Formula	Peak area
Control + 0	33.36	9,10-Anthracenedicarbonitrile	C_16_H_8_N_2_	3.32
	30.23	n-Octadecanol	C_18_H_38_O	3.11
	12.79	9-Octadecenamide	C18H35NO	72.91
MB246+ Pb100	39.28	Fumaric acid, 2-chlorophenyl 2,2,3,3-tetrafluoropropyl ester	C_13_H_9_ClF_4_O_4_	11.65
	38.53	9-Octadecenamide	C18H35NO	16.34
	34.73	Octadecanenitrile	C_18_H_35_N	11.47
	30.73	1-Phenanthrenol	C_14_H_10_O	8.17
MB322+ Pb 100	39.47	1-Tricosanol	C_23_H_48_O	20.66
	34.76	2-Fluoro-3-(trifluoromethyl)benzamide	C_8_H_5_F_4_NO	13.82
	35.36	1-Octadecene	C_18_H_36_	9.23
	31.48	2,3-Dibromonaphthalene	C_10_H_6_Br_2_	7.68
MB307+ Pb 100	38.58	6-Monoacetylmorphine	C_19_H_21_NO_4_	23.78
	35.38	Hexadecanamide	C_16_H_33_NO	21.05
	33.84	Octadecanenitrile	C_18_H_35_N	14.51
	30.08	9-Octadecenamide	C18H35NO	9.24
MB246+ Ni 100	35.66	Octadecanenitrile	C_18_H_35_N	12.11
	39.56	9-Octadecenamide	C18H35NO	17.28
MB322+ Ni 100	40.69	Fumaric acid, 2-chlorophenyl 2,2,3,3-tetrafluoropropyl ester	C_13_H_9_ClF_4_O_4_	19.68
	39.55	1-Heptadecanol, TMS	C_20_H_45_NSi	13.41
	18.21	2,3,6-Trifluoroaniline	C_6_H_4_F_3_N	3.27
MB307+ Ni 100	40.62	1-Tricosanol	C_23_H_48_O	6.06
	39.93	9-Octadecenamide	C18H35NO	14.87
MB246+ Cr 100	40.68	Fumaric acid, 2-chlorophenyl 2,2,3,3-tetrafluoropropyl ester	C_13_H_9_ClF_4_O_4_	21.91
	39.57	9-Octadecenamide	C18H35NO	18.75
MB322+ Cr 100	38.43	1-Heptadecanol, TMS	C_20_H_45_NSi	9.41
	35.37	9-Octadecene, (E)-	C_18_H_36_	9.14
	17.75	L-Glutamic acid	C_5_H_9_NO_4_	4.14
MB307+ Cr 100	39.48	1-Tricosanol	C_23_H_48_O	25.58
	38.54	Amodiaquine	C_20_H_22_ClN_3_O	14.52
	31.17	n-Hexadecanoic acid	C_16_H_32_O_2_	8.29
	21.47	N-(4-Acetamido-2-methylphenyl)acetamide	C_11_H_14_N_2_O_2_	12.28

#### a. Lipophilic (non-polar layer)

**Fig 4:**
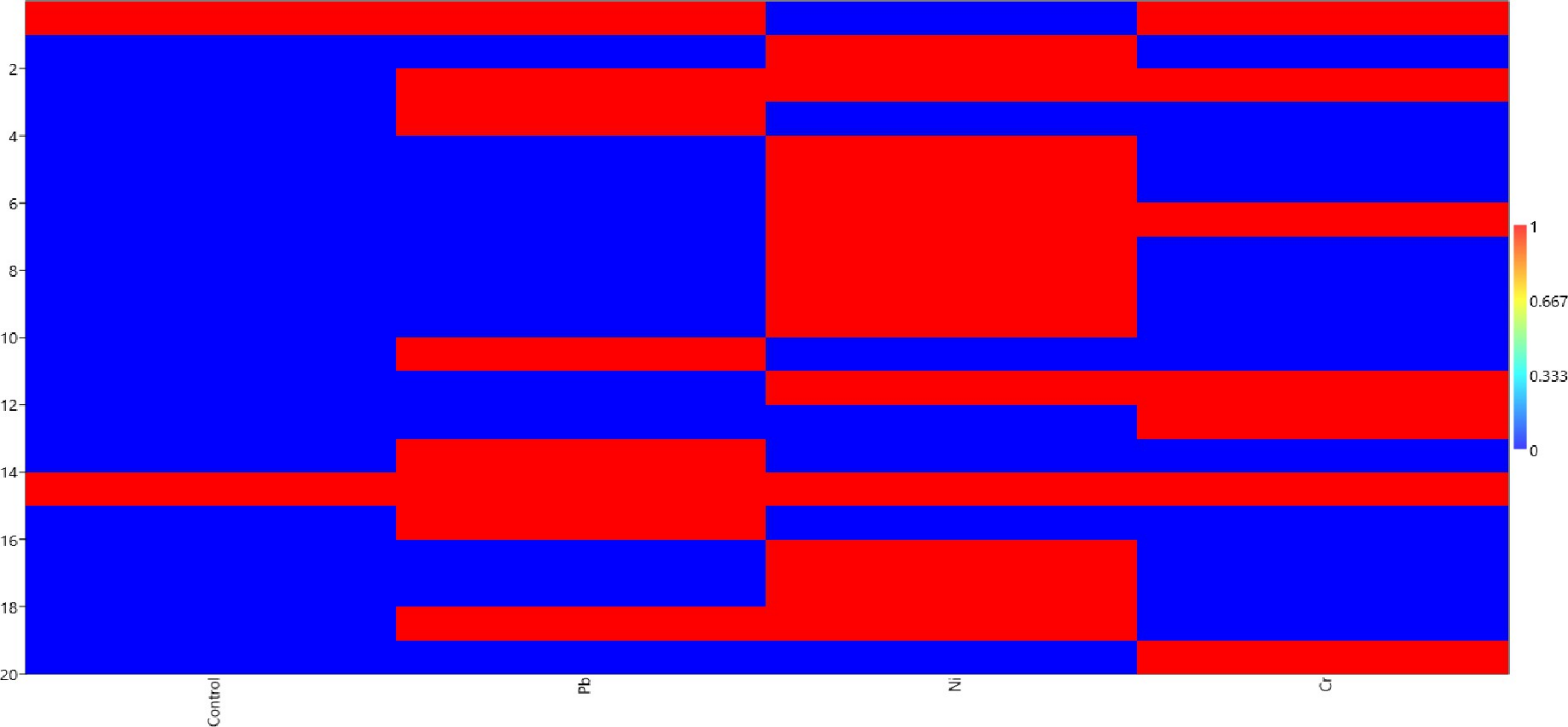
Heat map of metabolites produced in lipophilic layer.

GC analysis of *V*. *radiata* revealed up to 24 metabolites in lipophilic layer (Fig 4). 6-octadecenoic acid, methyl ester (12.87%) was major compound detected in lipophilic layer of MB322 inoculated seedlings under Pb stress. In the presence of Ni, MB307 inoculation resulted in maximum production of Glycine, N-(phenylacetyl)-, methyl ester (54.63%), whereas under Cr stress L-Glutamic acid was identified with maximum peak area (57.8%) in MB246 inoculated seedlings.

It was observed that only Picolinamide; Nonadecanoic acid; L-Cysteine and N,S-bis(2,6-difluorobenzoyl)-, methyl ester were produced when plant was under Pb stress whereas, Docosanoic acid; Cyclopropanecarboxylic acid,-2-(2-propynyl) methyl ester; Hexadecanamide; Picrolonic acid; 8-Methyl-6-nonenamide; 4-Dimethylamino-3,5-dinitrobenzoic acid and Glycolic acid, 2TMS derivative were particularly produced under Ni stress. In the presence of Cr, Nickel, (eta-2-2-diallyl ether); Tetradecanamide and cis-5,8,11-Eicosatrienoic acid, methyl ester was produced unlike other treatments. Hexadecanoic acid, methyl ester or pentadecanoic acid methyl ester were commonly produced in control and under all metals i.e. Pb, Ni and Cr (S1 Table in S1 File).

#### b. Methanolic (polar layer)

**Fig 5:**
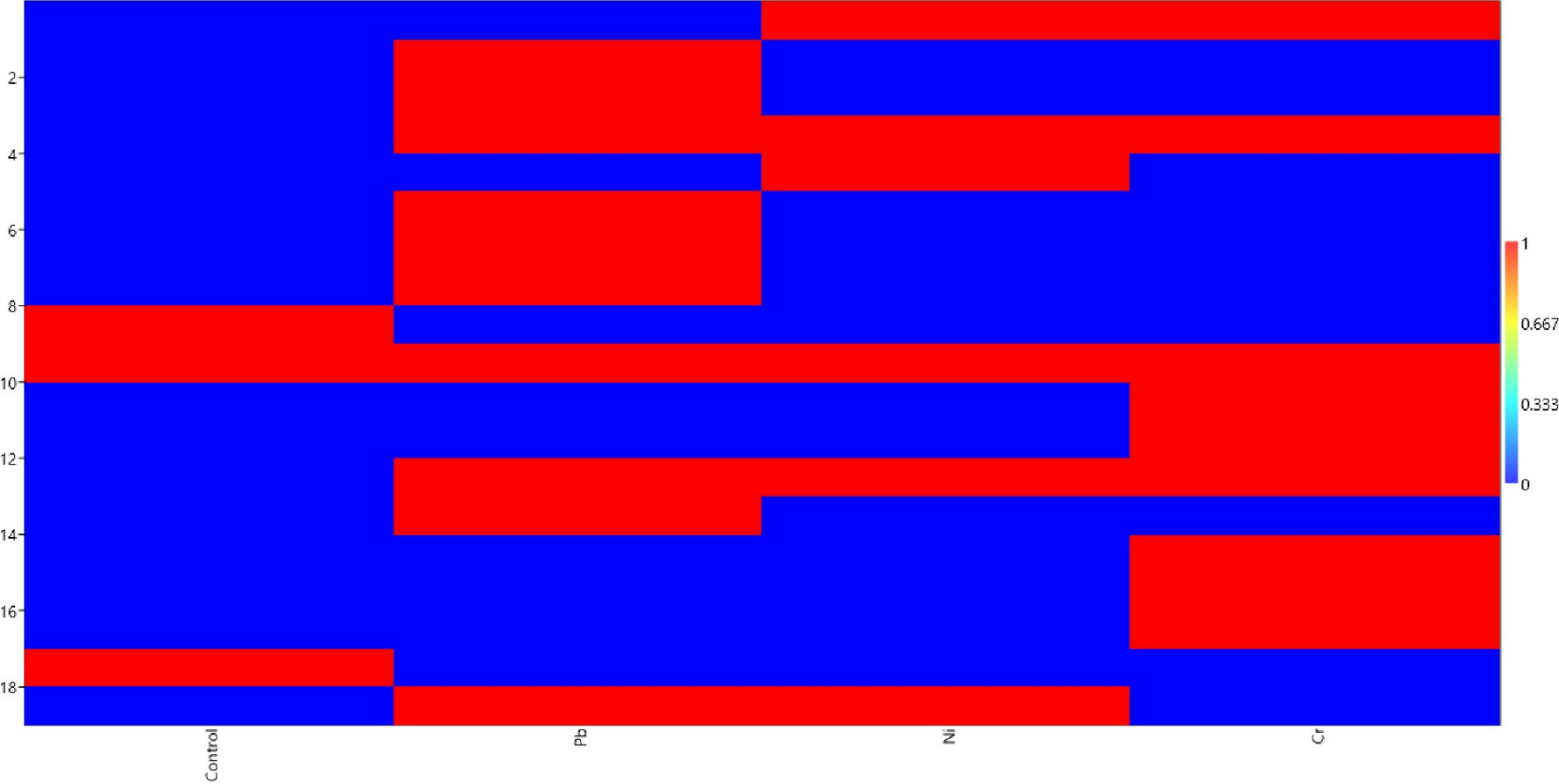
Heat map of metabolites produced in methanolic (polar layer).

In methanolic layer, total eighteen compounds were detected (Fig 5). Maximum concentration of compound detected in control sample was 9-Octadecenamide (72.91%). Whereas 6-Monoacetylmorphine was identified as highly secreted compound in Pb stress seedlings with MB307 inoculation. Under Ni stress, Fumaric acid, 2-chlorophenyl 2,2,3,3-tetrafluoropropyl ester were detected with highest concentration in MB322 inoculated seedlings. In Cr stressed seedlings,1-Tricosanol was identified as major metabolite (25.58%).

Comparison of metabolites under different metal stresses showed 9-Octadecenamide as commonly present compound in control and all metal treatments (S2 Table in S1 File). Fumaric acid ester and 1-Tricosanol was produced under all treatments (Pb, Ni, Cr). Metabolites found only under Pb stress were Hexadecanamide; 2-Fluoro-3-(trifluoromethyl) benzamide; 6-Monoacetylmorphine; 1-Phenanthrenol and 2,3-Dibromonaphthalene. The compound observed specifically under Ni stress was 2,3,6-Trifluoroaniline. Whereas n-Hexadecanoic acid; N-(4-Acetamido-2-methylphenyl) acetamide; Amodiaquine and L-Glutamic acid were produced particularly under Cr stress.

## Discussion

Phytoremediation is a green technology for the removal of heavy metal contaminants. Mung bean has been reported as an efficient plant for phytoremediation of various contaminants [41–43]. Phytoremediation efficiency of hyper-accumulating plants can further be improved by using metal resistant PGPB that alleviated metal stress and improved plant growth [44, 45]. Gupta [46] reported Pseudomonas *sp*. for successful phytoremediation.

In our study, metal resistant PGP strains *Bacillus pumilus* (MB246), *Serratia nematodiphila* (MB307) *and Delftia Lacustris* (MB322) were tested for bacterial assisted phytoremediation of Pb, Ni and Cr polluted soil by *Vigna radiata*. Growth of inoculated plants appeared healthy in metal stress soil as compared to uninoculated control. Improved growth under metal stress condition might be attributed towards PGP activities of these strains. Our results are in accordance with some other studies which reported enhanced growth of inoculated *Vigna radiata* in the presence of metals [47, 48]. Plants respond to abiotic stressors through activation of stress response genes. Expression of these genes increase by PGPB [49].

The Translocation factor and Bioaccumulation factors are two key indicators that determine the phytoremediation efficiency of plant. Major accumulation of metals in plant roots is one of the strategies used by plants to tolerate heavy metal stress. Metals are translocated from roots to shoot by membrane metal transporters and metal-binding proteins. Through certain mechanisms a balance is maintained between having enough essential metals available for metabolic functions and at the same time avoiding toxicity and to keep nonessential metals below their toxicity thresholds. Therefore excess amount of metals are stored in where they are less toxic for cellular metabolism e.g. in roots. Roots are expected to take up a greater amount of contaminants, and they represent a significant metal sink [50].

In current experiment, higher BF and low TF values for Cr showed that Cr retained more in roots as compared to shoots which indicated presence of plant mechanisms that prevent Cr translocation to shoots. Prapagdee et al. [51] also reported high BF and Low TF values for Cd by *Vigna radiata*. A study by Anjum et al. [52] presented *Vigna radiata* as good Cd stabilizer and Cd extractor. In our study, lowest BF values with highest TF values were obtained for Pb and Ni that indicated more translocation of Pb and Ni in shoot. This also relates to maximum metal removal of Pb from soil followed by Ni and Cr. According to Kumar et al. [53], Pb translocation from root to shoot is generally low in plants. However, in hyper-accumulating plants a higher TF is important.

Results depicted stimulatory effect of PGPB on metal extraction and translocation ability of *Vigna radiata* as observed with greater BF and TF values in most of the inoculated plants. This might be attributed towards increased bioavailability of metals through the synthesis of chelators and siderophores to solubilize and sequester metals from soil [54]. Our observations for improved phytoremediation with selected PGPB related to findings in our previous study under *in vitro* conditions. Moreover, use of *Serratia sp*. *Pseudomonas sp*. and *Bacillus pumilus* for improved phytoremediation of HM contaminated soil has been recognized in several studies [55–57].

Distribution of metals in different parts of plant exhibited maximum metal content in roots followed by stems and leaves. Maximum metal accumulation in roots is a consequence of metal tolerance by plant to reduce the effect of metal toxicity [58]. In current study, presence of higher Pb content in leaf relative to stem attributed towards high availability of Pb in the soil. Aransiola et al. [59] reported more accumulation of Pb in above ground biomass of *G*. *max L*. in the order of seeds> leaves> root>stem.

Uptake of mineral nutrients like, Ca, P, K, Mg, Mn, Na Fe, B and Zn is severely retarded in metal stress plants [32, 60]. Results of elemental analysis revealed Ca and K as highly abundant minerals, whereas S was present in minimum quantity. The elemental composition of metal stress *V*. *radiata* is not effected in the presence of PGPB. Bacterial inoculations enhanced the elemental composition in most of the cases. Desai et al. [61] also reported enhanced uptake of nutrients in *V*. *radiata* inoculated with PGPB. Plant growth promoting bacteria help plant hosts in the absorption of mineral nutrients in metal polluted soils [62]. *Serratia sp*. inoculation to linseed under Cd stress maintains an optimum level of nutrients [55]. Inoculation of *Pseudomonas sp*. enhanced nutrient uptake in sunflower and tomato [45]. Elemental concentration also varied in different parts of plant as depicted in results of current experiment. Ashraf et al. [63] reported higher concentrations of all the metals in the roots except for K, which was greater in the shoots of mung bean.

The exposure to heavy metal toxicity generates wide-ranging metabolic and physiological changes in plants [16]. Plants can cope with these heavy metal stresses by secondary metabolites production. Several plant secondary metabolites such as ascorbic acid, lipoic acid, α-tocopherol, and melatonin can be applied to improve tolerance to abiotic stress [64]. Plant growth promoting bacteria can contribute to the productions of plant secondary metabolites. Plants can detect certain molecules released by microbiomes through a chemical recognition system, which activate the plants to generate signal transduction networks and make corresponding changes in related gene activities, leading to the production of certain plant secondary metabolites. Horizontal gene transfer in plants-endophytes may also lead to changes in plant secondary metabolites [65].

In current study, various metabolites were identified through GC-MS in metal stress *Vigna radiata*. These metabolites belonged to various groups (organic acids, fatty acids, amino acids, amines, phenol, steroids etc) that might play a vital role in metal resistance and accumulation. Results of metabolic analysis revealed varied metabolic response of plants towards each metal as different heavy metals have different sites of action within the plant. Manipulation of genes responsible for production and action of secondary metabolites can improve the tolerance level and adaptability of plants under stress conditions [3]. The differences and separation of metabolites under each metal treatment was studied through principal component analysis (PCA).

**Fig 6:**
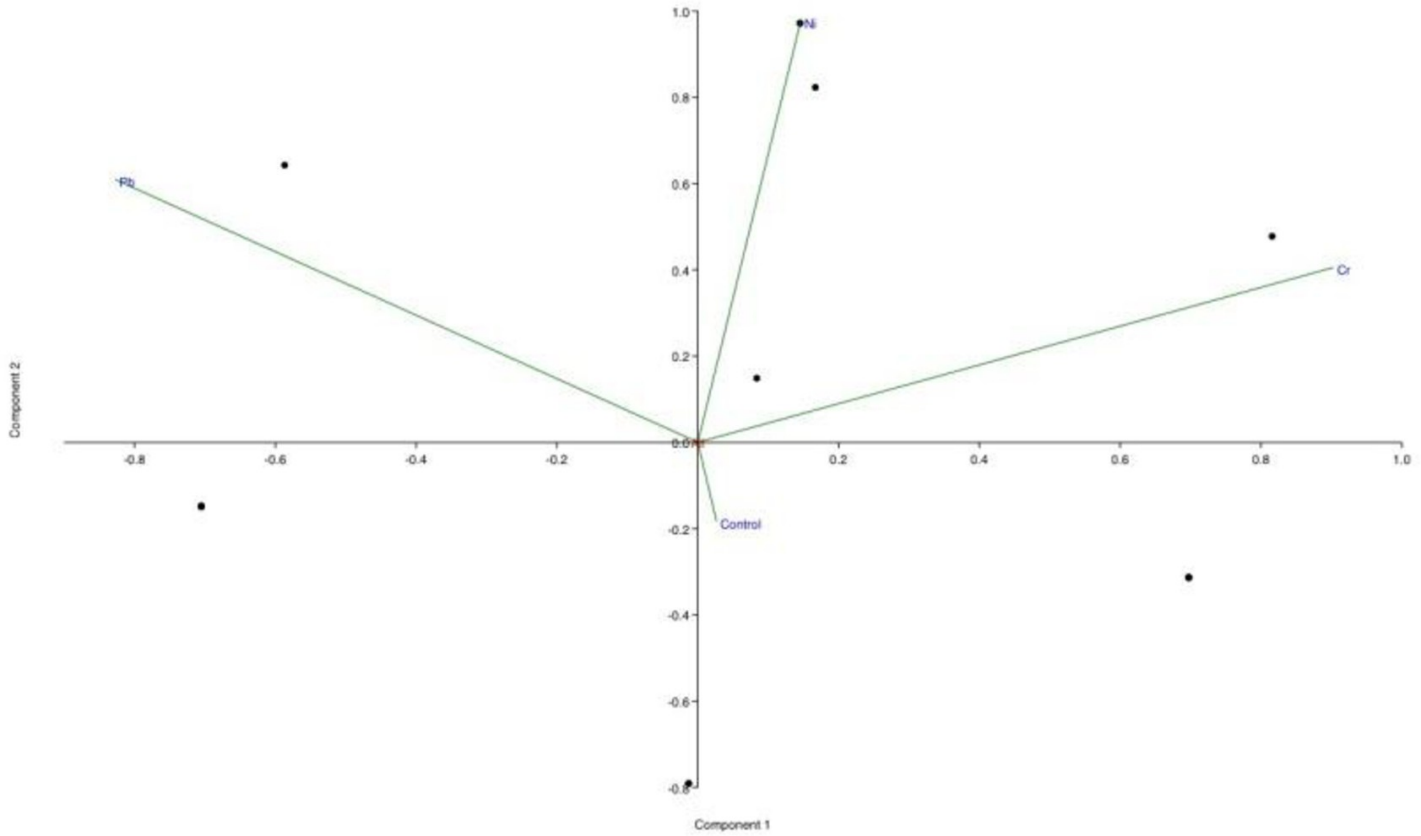
PCA biplot of metabolites in polar layer of *Vigna radiata*.

Majority of metabolites in polar layer were absent in control sample. However, most of them appeared in samples treated with different metals. Principal component analysis (PCA) of metabolites (Fig 6) showed that control and sample treated with Cr are closely related and produced few similar metabolites. Similarly, PCA biplot also revealed that Ni and Pb treated samples are somehow related to each other and produced few similar metabolites e.g. Octadecane nitrile. 1-Heptadecanol, TMS was found only in Ni and Cr stress seedlings.

**Fig 7:**
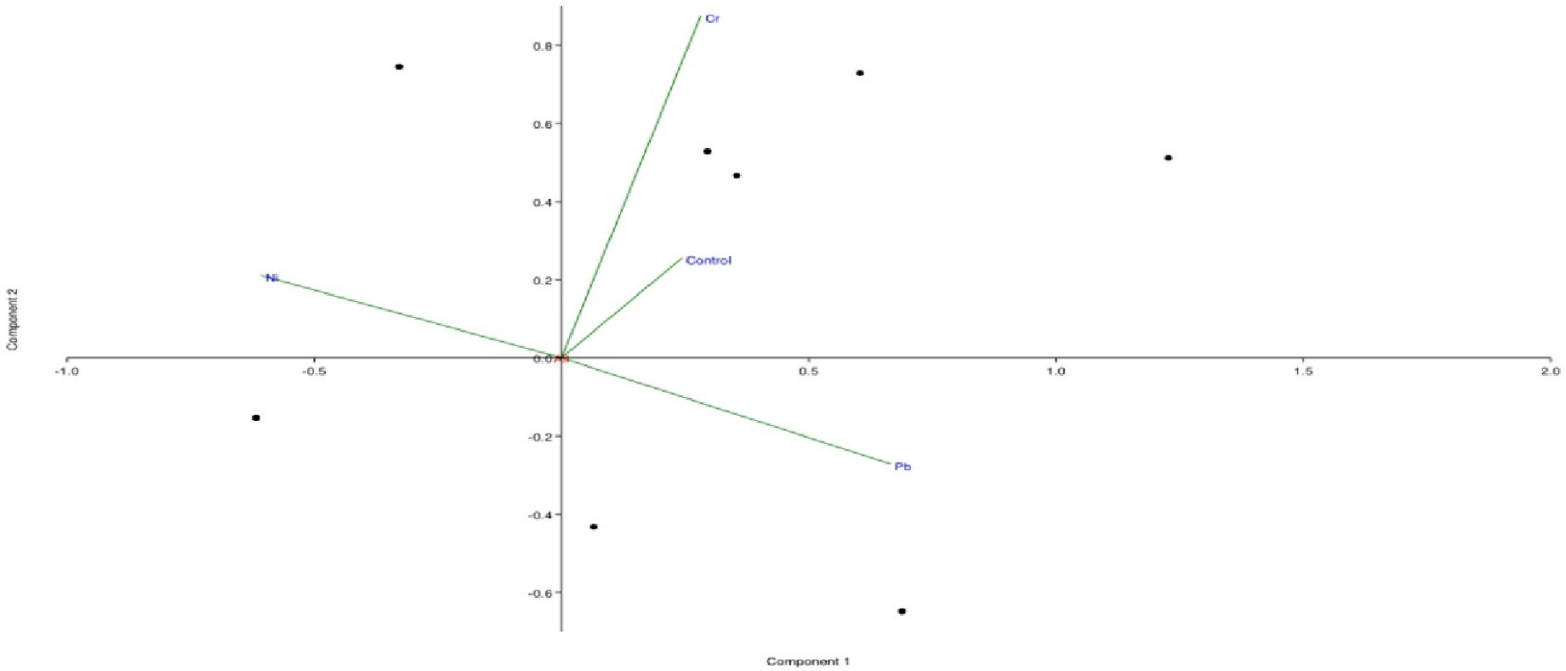
PCA biplot of metabolites in lipophilic layer of *Vigna radiata*.

In lipophilic layer, the metabolites detected in control were different from other samples. However, most of them appeared in samples treated with Ni followed by Pb and Cr. PCA biplots (Fig 7) showed that control and sample treated with Cr are closely related and produced few similar metabolites. Lead and Cr also produced few similar metabolites e.g. Eicosatrienoic acid, methyl ester. Metabolites produced under Ni stress are different from those produced under lead and chromium stress (Docosanoic acid, hexadeccaanamide etc). Octadecenoic acid methyl ester was identified in all metal stress (Pb, Ni and Cr). While 11-Octadecenoic acid, methyl ester was detected only in Cr stress seedlings. 9- octadecenoic acid has antimicrobial activity [66]. Azar et al. [67] reported fatty acid compounds; n-hexadecanoic acid, 9-octadecenoic acid (Z)- and octadecanoic acid found abundantly in *Leucobryum javense*. In a study by [20] organic acid like hexadecanoic acid, linoleic acid, α -linolenic acid and octadecanoic acid were significantly increased in response to Pb stress. Amino acids and derivatives can chelate metals conferring in plants resistant to toxic levels of HMs [68]. Amino acids like proline produced under heavy metals exposure effects the synthesis and activity of the antioxidant enzymes catalase, peroxidase. Some studies have reported for Ni complexation with phosphate, histidine and phytate [25]. In *Vinca rosea* the alleviated Ni stress levels attributed towards the enhanced synthesis of flavonoids, protein, proline and phenols [69].

## Conclusion

The current findings confirm that the application of PGPB is effective for phytoremediation of Pb, Ni and Cr polluted soil by *V*. *radiata*. The uptake of metals and its transport in various parts of plant with least accumulation in shoots make them safe for eating purpose keeping in view that the accumulated metal is not able to transport in the extreme upper part of plant shoot where grains are present. GC-MS analysis demonstrated a range of compounds (carbohydrates, fatty acids, amino acids, etc) produced distinctly under each metal stress which might be associated with metal tolerance ability of *V*. *radiata*. In future, further insight into direct relation of these metal responsive metabolites with metal tolerance is required at molecular and cellular level to investigate the mechanism of plant adaptation and genes involved in it which will give further advancement in gene manipulation of plant and bacteria for efficient phytoremediation.

## Supporting information

S1 File (DOCX)

## References

[1] Afegbua SL, Batty LC. Effect of plant growth promoting bacterium; *Pseudomonas putida* UW4 inoculation on phytoremediation efficacy of monoculture and mixed culture of selected plant species for PAH and lead spiked soils. Int. J. Phytoremediation. 2019; 21(3): 200–208. 30656952 10.1080/15226514.2018.1501334

[2] Ren X, Guo S, Tian W, Chen Y, Han H, Chen E, et al. Effects of plant growth-promoting bacteria (PGPB) inoculation on the growth, antioxidant activity, Cu uptake, and bacterial community structure of rape (*Brassica napus* L.) grown in Cu-contaminated agricultural soil. Front. Microbiol. 2019; 10: 1455. 31316489 10.3389/fmicb.2019.01455 PMC6610483

[3] Jan R, Asaf S, Numan M, Kim KM. Plant Secondary metabolite biosynthesis and transcriptional regulation in response to biotic and abiotic stress conditions. Agron. J. 2021; 11 (5): 968.

[4] Pattnaik S, Dash D, Mohapatra S, Pattnaik M, Marandi AK, Das S, et al. Improvement of rice plant productivity by native Cr (VI) reducing and plant growth promoting soil bacteria *Enterobacter cloacae*. Chemosphere. 2020; 240: 124895. 31550588 10.1016/j.chemosphere.2019.124895

[5] Jabeen Z, Irshad F, Habib A, Hussain N, Sajjad M, Mumtaz S, et al. Alleviation of cadmium stress in rice by inoculation of *Bacillus cereus*. Peer J. 2022; 10: e13131. 35529485 10.7717/peerj.13131 PMC9070326

[6] Jach M. E., Sajnaga E., and Ziaja M. (2022). Utilization of Legume-Nodule Bacterial Symbiosis in Phytoremediation of Heavy Metal-Contaminated Soils. Biology, 11(5), 676. doi: 10.3390/biology11050676 35625404 PMC9138774

[7] Pajuelo E, Rodríguez-Llorente ID, Lafuente A, Caviedes MÁ. Legume–rhizobium symbioses as a tool for bioremediation of heavy metal polluted soils. In: Biomanagement of metal-contaminated soils. Springer. 2011; pp. 95–123. Dordrecht.

[8] Ahmad MSA, Hussain M, Samina Alvi AK. Photosynthetic performance of two mung bean (*Vigna radiata*) cultivars under lead and copper stress. Int. J. Agric. Biol. 2008; 10: 167–172.

[9] Hassan M, Mansoor S. Oxidative stress and antioxidant defense mechanism in mung bean seedlings after lead and cadmium treatments Turk. J. Agric. 2014; For 38(1); 55–61.

[10] Tao L, Guo M, Ren J. Effects of cadmium on seed germination, coleoptile growth, and root elongation of six pulses. Pol. J. Environ. Stud. 2015; 24(1): 295–299.

[11] Jabeen N, Abbas Z, Iqbal M, Rizwan M, Jabbar A, Farid M, et al. Glycinebetaine mediates chromium tolerance in mung bean through lowering of Cr uptake and improved antioxidant system. Arch. Agron. Soil Sci. 2016; 62(5): 648–662.

[12] Mahawar L, Popek R, Shekhawat GS, Alyemeni MN, Ahmad P. Exogenous hemin improves Cd2+ tolerance and remediation potential in *Vigna radiata* by intensifying the HO-1 mediated antioxidant defence system. Scientific reports. 2021; 11(1): 2811. 33531561 10.1038/s41598-021-82391-1 PMC7854669

[13] Saif S, and Khan M.S. Assessment of heavy metals toxicity on plant growth promoting rhizobacteria and seedling characteristics of Pseudomonas putida SFB3 inoculated greengram. Acta Sci. Agric. 2017; 1: 47–56.

[14] Rajendran SK, Sundaram L. Degradation of heavy metal contaminated soil using plant growth promoting rhizobacteria (PGPR): Assess their remediation potential and growth influence of *Vigna radiata*. L. Int. J. Agricl. Tech. 2020; 16(2): 365–376.

[15] Sun Y, Wu J, Shang X, Xue L, Ji G, Chang S, et al. Screening of Siderophore-Producing Bacteria and Their Effects on Promoting the Growth of Plants. Currnt Microbiol. 2022; 79(5): 1–12. 10.1007/s00284-022-02777-w 35396958

[16] Gill M. Heavy metal stress in plants: a review. Int. J. Adv. Res. 2014; 2(6): 1043–1055.

[17] Luo Q, Wang S, Sun LN, Wang H. Metabolic profiling of root exudates from two ecotypes of *Sedum alfredii* treated with Pb based on GC-MS. Scientific reports. 2017; 7(1): 1–9. 28051189 10.1038/srep39878 PMC5209681

[18] Anjitha KS, Sameena PP, Jos Puthur T. Functional aspects of plant secondary metabolites in metal stress tolerance and their importance in pharmacology. Plant Stress. 2 (2021) 100038.

[19] Jayanthy V, Geetha R, Rajendran R, Prabhavathi P, Sundaram SK, Kumar SD, et al. Phytoremediation of dye contaminated soil by *Leucaena leucocephala* (subabul) seed and growth assessment of *Vigna radiata* in the remediated soil. Saudi J. Biol. Sci. 2014; 21(4): 324–333. 25183943 10.1016/j.sjbs.2013.12.001 PMC4150223

[20] Pandey VK. Speciation and distribution of heavy metals in plants. J. Crop Sci. Res. 2017; 2(1): 1–9.

[21] Wang Y, Xu L, Shen H, Wang J, Liu W, Zhu X, et al. Metabolomic analysis with GC-MS to reveal potential metabolites and biological pathways involved in Pb and Cd stress response of radish roots. Sci. Rep. 2015; 5: 18296. 26673153 10.1038/srep18296 PMC4682141

[22] Xie Y, Ye S, Wang Y, Xu L, Zhu X, Yang J, et al. Transcriptome-based gene profiling provides novel insights into the characteristics of radish root response to Cr stress with next-generation sequencing. Front. Plant Sci. 2015; 6: 202. doi: 10.3389/fpls.2015.00202 25873924 PMC4379753

[23] Debela F, Arocena JM, Thring RW, Whitcombe T. Organic acid-induced release of lead from pyromorphite and its relevance to reclamation of Pb-contaminated soils. Chemosphere. 2010; 80(4): 450–456. doi: 10.1016/j.chemosphere.2010.04.025 20444487

[24] Sharma B, Kothari R, Singh RP. Growth performance, metal accumulation and biochemical responses of Palak (*Beta vulgaris* L. var. Allgreen H-1) grown on soil amended with sewage sludge-fly ash mixtures. Environ. Sci. Pollut. Res. 2018; 25(13): 12619–12640. 10.1007/s11356-018-1475-7 29468393

[25] Van Der Ent A, Callahan DL, Noller BN, Mesjasz-Przybylowicz J, Przybylowicz WJ, Barnabas A, et al. Nickel biopathways in tropical nickel hyperaccumulating trees from Sabah (Malaysia). Sci. Rep. 2017; 7: 41861. doi: 10.1038/srep41861 28205587 PMC5311975

[26] Xie Y, Hu L, Du Z, Sun X, Amombo E, Fan J, et al. Effects of cadmium exposure on growth and metabolic profile of Bermuda grass [*Cynodon dactylon* (L.) Pers.]. PloS One. 2014; 9(12): 115279. 10.1371/journal.pone.0115279 PMC4278907 25545719

[27] Amtmann A, Hammond JP, Armengaud P, White PJ. Nutrient sensing and signalling in plants: potassium and phosphorus. Adv. Bot. Res. 2005; 43: 209–257.

[28] Li S, Yang W, Yang T, Chen Y, Ni W. Effects of cadmium stress on leaf chlorophyll fluorescence and photosynthesis of *Elsholtzia argyi*—a cadmium accumulating plant. Int. J. Phytoremediation. 2015; 17(1): 85–92. 25174428 10.1080/15226514.2013.828020

[29] De Britto AJ, and Raj MS TLS. Molecular response of Rice (*Oryza Sativa* L.) To Zinc stress by A. Joh De Britto, M.sutha and T. Leon Stephan Raj. Life Sci Leaflets. 2011; 15: 517-to.

[30] Lamhamdi M, El Galiou O, Bakrim A, Nóvoa-Muñoz JC, Arias-Estévez M, Aarab A, et al. Effect of lead stress on mineral content and growth of wheat (*Triticum aestivum*) and spinach (*Spinacia oleracea*) seedlings. Saudi J. Biol. Sci. 2013; 20(1): 29–36. 23961216 10.1016/j.sjbs.2012.09.001 PMC3730938

[31] Khermandar K, Mahdavi A, Ahmady AS. Differential expression of Lead accumulation during two growing seasons by desert shrub *Acacia victoriae* L. Desert. 2016; 21(2): 143–154.

[32] Ashraf MA, Hussain I, Rasheed R, Iqbal M, Riaz M, Arif MS. Advances in microbe-assisted reclamation of heavy metal contaminated soils over the last decade: a review. J. Environ. Manage. 2017; 198: 132–143. doi: 10.1016/j.jenvman.2017.04.060 28456029

[33] Gusain YS, Singh US, Sharma AK. Bacterial mediated amelioration of drought stress in drought tolerant and susceptible cultivars of rice (*Oryza sativa* L.). Af. J. Biotech, 2015; 14(9): 764–773.

[34] Jones JB. Laboratory guide for conducting soil tests and plant analysis. CRC press. 2001.

[35] Schulte EE, Hopkins BG. Estimation of soil organic matter by weight loss‐on‐ignition. Soil organic matter: Analysis and interpretation. 1996; 46: 21–31.

[36] Mussa SB, Elferjani HS, Haroun FA, Abdelnabi FF. Determination of available nitrate, phosphate and sulfate in soil samples. Int. J. Pharmtech. Res. 2009; 1(3): 598–604.

[37] Zulfiqar U. Bacterial assisted phytoremediation of selected pollutants. [Ph.D Dissertation]. Fatima Jinnah Women University Rawalpindi, Pakistan; 2019.

[38] Hewitt EJ. Mineral nutrition of plants in culture media. In: Plant Physiology. (Steward F.C. eds). Academic Press, pp New York, 1963; 99–137.

[39] Yadav SK, Juwarkar AA, Kumar GP, Thawale PR, Singh SK, Chakrabarti T. Bioaccumulation and phyto-translocation of arsenic, chromium and zinc by *Jatropha curcas* L.: impact of dairy sludge and biofertilizer. Bioresour. Technol. 2009; 100(20): 4616–4622. 19481929 10.1016/j.biortech.2009.04.062

[40] Sun X., Zhang J., Zhang H., Ni Y., Zhang Q., Chen J., et al., 2010. The responses of *Arabidopsis thaliana* to cadmium exposure explored via metabolite profiling. Chemosphere. 78(7), 840–5. 20044121 10.1016/j.chemosphere.2009.11.045

[41] Mathiyazhagan N, Natarajan D. Phytoremediation efficiency of edible and economical crops on waste dumps of Bauxite mines, Salem district, Tamil Nadu, India. In: On a Sustainable Future of the Earth’s Natural Resources (Ramkumar M. (ed)). 2013; Springer, Berlin, Heidelberg.

[42] Anjum NA, Umar S, Iqbal M. Assessment of cadmium accumulation, toxicity, and tolerance in *Brassicaceae* and *Fabaceae plants*—implications for phytoremediation. Environ. Sci. Pol. Res.2014; 21(17): 10286–10293. 10.1007/s11356-014-2889-5 24756685

[43] Maheshwari G, Setia K, Gauba Mathur S. Exploring phytoremediation potential for estrogen hormone. Int. J. Res. Rev. 2019; 6(9): 195–202.

[44] Raklami A, Oufdou K, Tahiri AI, Mateos-Naranjo E, Navarro-Torre S, Rodríguez-Llorente ID, et al. Safe cultivation of *Medicago sativa* in metal-polluted soils from semi-arid regions assisted by heat-and metallo-resistant PGPR. Microorganisms. 2019; 7(7): 212. 10.3390/microorganisms7070212 PMC6680742 31336693

[45] Pishchik V, Mirskaya G, Chizhevskaya E, Chebotar V, Chakrabarty D. Nickel stress-tolerance in plant-bacterial associations. Peer J. 2021; 9, e12230. doi: 10.7717/peerj.12230 34703670 PMC8487243

[46] Gupta P, Rani R, Chandra A, Kumar V. Potential applications of *Pseudomonas* sp. (strain CPSB21) to ameliorate Cr 6+ stress and phytoremediation of tannery effluent contaminated agricultural soils. Sci. Rep. 2018; 8(1): 1–10. 29559691 10.1038/s41598-018-23322-5 PMC5861048

[47] Chakraborty S, Das S, Banerjee S, Mukherjee S, Ganguli A, Mondal S. Heavy metals bio-removal potential of the isolated *Klebsiella* Sp TIU20 strain which improves growth of economic crop plant (*Vigna radiata* L.) under heavy metals stress by exhibiting plant growth promoting and protecting traits. Biocatal. Agric. Biotechnol. 2021; 38: 102204.

[48] Subrahmanyam G, Sharma RK, Kumar GN, Archana G. *Vigna radiata* var. GM4 plant growth enhancement and root colonization by a multi-metal-resistant plant growth-promoting bacterium *Enterobacter* sp. C1D in Cr (VI)-amended soils. Pedosphere. 2018; 28(1): 144–156.

[49] Begum N, Hu Z, Cai Q, Lou L. Influence of PGPB inoculation on HSP70 and HMA3 gene expression in switchgrass under cadmium stress. Plants. 2019; 8(11): 504. doi: 10.3390/plants8110504 31739628 PMC6918137

[50] Maria De & Rivelli A.R. Trace element accumulation and distribution in sunflower plants at the stages of flower bud and maturity. Italian Journal of Agronomy. 2013; 8(1), e9–e9.

[51] Prapagdee S, Piyatiratitivorakul S, Petsom A, Tawinteung N. Application of biochar for enhancing cadmium and zinc phytostabilization in *Vigna radiata* L. cultivation. Water Air Soil Pollut. 2014; 225(12): 2233.

[52] Anjum NA, Umar S, Iqbal M. Assessment of cadmium accumulation, toxicity, and tolerance in Brassicaceae and Fabaceae plants—implications for phytoremediation. Environ. Sci. Pollut. Res, 2014; 21(17): 10286–10293. 10.1007/s11356-014-2889-5 24756685

[53] Kumar N, Dushenkov V, Motto H, Raskin I. Phytoextraction: the use of plants to remove heavy metals from soils. Environ. Sci. Techn. 1995; 29: 1232–1238. doi: 10.1021/es00005a014 22192016

[54] Braud A, Jézéquel K, Lebeau T. Impact of substrates and cell immobilization on siderophore activity by *Pseudomonads* in a Fe and/or Cr, Hg, Pb containing-medium. J. Hazard. Mater. 2007; 144(1–2): 229–239. 17112663 10.1016/j.jhazmat.2006.10.014

[55] De-Bashan LE, Hernandez JP, Bashan Y, and Maier RM. *Bacillus pumilus* ES4: candidate plant growth-promoting bacterium to enhance establishment of plants in mine tailings. Environ. Exp. Bot. 2010; 69(3): 343–352. doi: 10.1016/j.envexpbot.2010.04.014 25009362 PMC4084739

[56] Montalban B, Thijs S, Lobo M, Weyens N, Ameloot M, Vangronsveld J, et al. Cultivar and metal-specific effects of endophytic bacteria in *Helianthus* tuberosus exposed to Cd and Zn. Int. J. Mol. Sci. 2017; 18(10): 2026. 10.3390/ijms18102026 PMC5666708 28934107

[57] Shahid M, Javed MT, Masood S, Akram MS, Azeem M, Ali Q, et al. *Serratia* sp. CP‐13 augments the growth of cadmium (Cd)‐stressed Linum usitatissimum L. by limited Cd uptake, enhanced nutrient acquisition and antioxidative potential. J. Appl. Microbiol. 2019; 126(6): 1708–1721. 30882965 10.1111/jam.14252

[58] Yurekli F, Porgali Z. The effects of excessive exposure to copper in bean plants. Acta Biologica Cracoviensia Series Botanica. 2006; 48(2): 7–13.

[59] Aransiola SA, Ijah UJJ, Abioye OP. Phytoremediation of lead polluted soil by *Glycine max* L. Appl. Environ. Soil Sci. 2013; (7).

[60] Shamsi IH, Wei K, Jilani G, Zhang GP. Interactions of cadmium and aluminum toxicity in their effect on growth and physiological parameters in soybean. J. Zhejiang Univ. Sci. B. 2007; 8(3): 181–188. doi: 10.1631/jzus.2007.B0181 17323430 PMC1810381

[61] Desai S, Mistry J, Shah F, Chandwani S, Amaresan N, Supriya NR. Salt-tolerant bacteria enhance the growth of mung bean (*Vigna radiata* L.) and uptake of nutrients, and mobilize sodium ions under salt stress condition. Int J Phytoremediation 2022; 6:1–8. 10.1080/15226514.2022.2057419 35382669

[62] Zubair M, Shakir M, Ali Q, Rani N, Fatima N, Farooq S, et al. Rhizobacteria and phytoremediation of heavy metals. Environ. Technol. Reviews. 2016; 5(1): 112–119.

[63] Ashraf MY, Roohi M, Iqbal Z, Ashraf M, Ozturk M, Gucel S. Cadmium (Cd) and Lead (Pb) induced inhibition in growth and alteration in some biochemical attributes and mineral accumulation in Mung bean [*Vigna radiata* (L.) Wilczek]. Commun. Soil Sci. Plant. 2015; Anal Taylor and Francis (ISSN: 0010-3624 (Print), 1532–2416).

[64] Godoy Francisca et al. “Abiotic Stress in Crop Species: Improving Tolerance by Applying Plant Metabolites.” Plants. 2021; 10: 2 186. doi: 10.3390/plants10020186 33498148 PMC7908993

[65] Pang Z, Chen J, Wang T, Gao C, Li Z, Guo L, et al. Linking plant secondary metabolites and plant microbiomes: a review. Front. Plant Sci. 2021; 12: 621276. doi: 10.3389/fpls.2021.621276 33737943 PMC7961088

[66] Ghavam M, Manconi M, Manca ML, Bacchetta G. Extraction of essential oil from *Dracocephalum kotschyi Boiss*.(Lamiaceae), identification of two active compounds and evaluation of the antimicrobial properties. J. Ethnopharmacol. 2021; 267: 113513. 33172599 10.1016/j.jep.2020.113513

[67] Azar AWP, Rosleine D, Faizal A. Secondary metabolite profiles in the methanolic extract of Leucobryum javense isolated from tropical montane forest in West Java, Indonesia. In AIP Conference Proceedings. 2019; 2120 (1): p. 030027. AIP Publishing LLC.

[68] Kumar A, Thakur N. Phytoremediation: Green technology for heavy metal clean up from contaminated soils. Int. J. Chem. Stud. 2019; 7(5): 1987–1994.

[69] Khan WU, Ahmad SR, Yasin NA, Ali A, Ahmad A, Akram W. Application of *Bacillus megaterium* MCR-8 improved phytoextraction and stress alleviation of nickel in *Vinca rosea*. Int. J. Phytoremediation. 2017; 19(9): 813–824. 28699781 10.1080/15226514.2017.1290580

