# Supplementary material for: Metabolites produced by inoculated Vigna radiata during bacterial assisted phytoremediation of Pb, Ni and Cr polluted soil
Source: PLoS One. 2022 Nov 10;17(11):e0277101. doi: 10.1371/journal.pone.0277101 (PMC9648758; doi:10.1371/journal.pone.0277101)
Supplement: S1 File — (DOCX) [file pone.0277101.s001.docx]

**Metabolites produced by *Vigna radiata* during bacterial assisted phytoremediation of Pb, Ni and Cr.**

Uzma Zulfiqar^1*^, Azra Yasmin ^1&2^ and Anila Fariq^2^.

*^1*^ Department of Environmental Sciences, Fatima Jinnah Women University, Rawalpindi, Pakistan*

*^2^ Department of Biotechnology, Fatima Jinnah Women University, Rawalpindi, Pakistan*

*Corresponding author E- mail: [uzma21awan@gmail.com](mailto:uzma21awan@gmail.com) and azrayasmin@fjwu.edu.pk

**Supplementary Tables.**

**S1 Table.** Physiochemical features of soil

| **Soil Parameters** | **Values** |
| --- | --- |
| **Colour** | Light brown |
| **Texture** | Sandy- loam |
| **Electrical conductivity (µs/cm)** | 144 |
| **pH** | 9.0 |
| **Organic matter (%)** | 2.11 |
| **Moisture (%)** | 1.62 |
| **Phosphates (mg/kg)** | 54.78 |
| **Sulphates (mg/kg)** | 0.5 |

**S2 Table.** Comparison of compounds produced in lipohilic (non polar) layer of uninoculated and inoculated *Vigna radiata* seedlings.

| **Compound** | **Control** | **Pb** | **Ni** | **Cr** |
| --- | --- | --- | --- | --- |
| Eicosatrienoic acid, methyl ester | + | + |  | + |
| 8-Methyl-6-nonenamide |  |  | + |  |
| Octadecenoic acid, methyl ester |  | + | + | + |
| cis-13-Eicosenoic acid, picolinyl ester |  | + |  |  |
| Cyclopropanecarboxylic acid,-2-(2-propynyl) methyl ester |  |  | + |  |
| Docosanoic acid |  |  | + |  |
| Glycine, N-(phenylacetyl)-, methyl ester |  |  | + | + |
| Glycolic acid, 2TMS derivative |  |  | + |  |
| Hexadecanamide |  |  | + |  |
| 4-Dimethylamino-3,5-dinitrobenzoic acid |  |  | + |  |
| l-Cysteine, N,S-bis(2,6-difluorobenzoyl)-, methyl ester |  | + |  |  |
| L-Glutamic acid |  |  | + | + |
| Nickel, (eta-2-2-diallyl ether) |  |  |  | + |
| Nonadecanoic acid |  | + |  |  |
| Pentadecanoic acid | + | + | + | + |
| Picolinamide |  | + |  |  |
| Picrolonic acid |  |  | + |  |
| Sarcosine, N-(2-trifluoromethylbenzoyl)-, butyl ester |  |  | + |  |
| Sarcosine, N-(3-bromobenzoyl)-, butyl ester |  | + | + |  |
| Tetradecanamide |  |  |  | + |

**S3 Table.** Comparison of compounds produced in methanolic (polar) layer of uninoculated and inoculated *Vigna radiata* seedlings.

| **Compound** | **Control** | **Pb** | **Ni** | **Cr** |
| --- | --- | --- | --- | --- |
| 1-Heptadecanol, TMS |  |  | + | + |
| 1-Octadecene |  | + |  |  |
| 1-Phenanthrenol |  | + |  |  |
| 1-Tricosanol |  | + | + | + |
| 2,3,6-Trifluoroaniline |  |  | + |  |
| 2,3-Dibromonaphthalene |  | + |  |  |
| 2-Fluoro-3-(trifluoromethyl)benzamide |  | + |  |  |
| 6-Monoacetylmorphine |  | + |  |  |
| 9,10-Anthracenedicarbonitrile | + |  |  |  |
| 9-Octadecenamide | + | + | + | + |
| 9-Octadecene, (E)- |  |  |  | + |
| Amodiaquine |  |  |  | + |
| Fumaric acid, 2-chlorophenyl 2,2,3,3-tetrafluoropropyl ester |  | + | + | + |
| Hexadecanamide |  | + |  |  |
| L-Glutamic acid |  |  |  | + |
| N-(4-Acetamido-2-methylphenyl)acetamide |  |  |  | + |
| n-Hexadecanoic acid |  |  |  | + |
| n-Octadecanol | + |  |  |  |
| Octadecanenitrile |  | + | + |  |

**S4 Table. Metabolites identified in lipophilic (non polar) layer of *V. radiata***

| **No** | **Compounds** | **Control** | **Pb** | **Ni** | **Cr** |
| --- | --- | --- | --- | --- | --- |
|  | Eicosatrienoic acid, methyl ester | 1 | 1 | 0 | 1 |
|  | 8-Methyl-6-nonenamide | 0 | 0 | 1 | 0 |
|  | Octadecenoic acid, methyl ester | 0 | 1 | 1 | 1 |
|  | cis-13-Eicosenoic acid, picolinyl ester | 0 | 1 | 0 | 0 |
|  | Cyclopropanecarboxylic acid,-2-(2-propynyl) methyl ester | 0 | 0 | 1 | 0 |
|  | Docosanoic acid | 0 | 0 | 1 | 0 |
|  | Glycine, N-(phenylacetyl)-, methyl ester | 0 | 0 | 1 | 1 |
|  | Glycolic acid, 2TMS derivative | 0 | 0 | 1 | 0 |
|  | Hexadecanamide | 0 | 0 | 1 | 0 |
|  | 4-Dimethylamino-3,5-dinitrobenzoic acid | 0 | 0 | 1 | 0 |
|  | l-Cysteine, N,S-bis(2,6-difluorobenzoyl)-, methyl ester | 0 | 1 | 0 | 0 |
|  | L-Glutamic acid | 0 | 0 | 1 | 1 |
|  | Nickel, (eta-2-2-diallyl ether) | 0 | 0 | 0 | 1 |
|  | Nonadecanoic acid | 0 | 1 | 0 | 0 |
|  | Pentadecanoic acid | 1 | 1 | 1 | 1 |
|  | Picolinamide | 0 | 1 | 0 | 0 |
|  | Picrolonic acid | 0 | 0 | 1 | 0 |
|  | Sarcosine, N-(2-trifluoromethylbenzoyl)-, butyl ester | 0 | 0 | 1 | 0 |
|  | Sarcosine, N-(3-bromobenzoyl)-, butyl ester | 0 | 1 | 1 | 0 |
|  | Tetradecanamide | 0 | 0 | 0 | 1 |

| **No** | **Polar layer** | control | Pb | Ni | Cr |
| --- | --- | --- | --- | --- | --- |
| 1 | 1-Heptadecanol, TMS | 0 | 0 | 1 | 1 |
| 2 | 1-Octadecene | 0 | 1 | 0 | 0 |
| 3 | 1-Phenanthrenol | 0 | 1 | 0 | 0 |
| 4 | 1-Tricosanol | 0 | 1 | 1 | 1 |
| 5 | 2,3,6-Trifluoroaniline | 0 | 0 | 1 | 0 |
| 6 | 2,3-Dibromonaphthalene | 0 | 1 | 0 | 0 |
| 7 | 2-Fluoro-3-(trifluoromethyl)benzamide | 0 | 1 | 0 | 0 |
| 8 | 6-Monoacetylmorphine | 0 | 1 | 0 | 0 |
| 9 | 9,10-Anthracenedicarbonitrile | 1 | 0 | 0 | 0 |
| 10 | 9-Octadecenamide | 1 | 1 | 1 | 1 |
| 11 | 9-Octadecene, (E)- | 0 | 0 | 0 | 1 |
| 12 | Amodiaquine | 0 | 0 | 0 | 1 |
| 13 | Fumaric acid, 2-chlorophenyl 2,2,3,3-tetrafluoropropyl ester | 0 | 1 | 1 | 1 |
| 1 | Hexadecanamide | 0 | 1 | 0 | 0 |
| 4 | L-Glutamic acid | 0 | 0 | 0 | 1 |
| 15 | N-(4-Acetamido-2-methylphenyl)acetamide | 0 | 0 | 0 | 1 |
| 16 | n-Hexadecanoic acid | 0 | 0 | 0 | 1 |
| 17 | n-Octadecanol | 1 | 0 | 0 | 0 |
| 18 | Octadecanenitrile | 0 | 1 | 1 | 0 |

**S5 Table. Metabolites identified in methanolic (polar) layer of *V. radiata***
